# Intergenerational Rural Health Programming Through Active Aging for L.I.F.E.

**DOI:** 10.1093/geroni/igaf122.835

**Published:** 2025-12-31

**Authors:** Emily Roberts, Xuewei Chen

**Affiliations:** Oklahoma State University, Stillwater, Oklahoma, United States; Oklahoma State University, Stillwater, Oklahoma, United States

## Abstract

Aging is a process in which everyone is involved, as it is a lifelong experience, not just a later life occurrence. The Active Aging for L.I.F.E. collaborative project provides a novel rural health education approach promoting positive health outcomes for intergenerational participants. The program is developed to focus on the interrelationships between Longevity, Independence, Fitness and Engagement (L.I.F.E.), bringing together L.I.F.E. Leader teams of older adults (50+) and college-age students who are trained in the four L.I.F.E. modules. The L.I.F.E. Leaders then take the program to rural high school students in four sessions, focusing on the interconnected network of personal and professional health and wellness opportunities. This presentation provides guidance and tools for the Active Aging for L.I.F.E. program and demonstrates through mixed-method findings the potential of intergenerational rural community-based active aging programming.

